# College students responding to the Chinese version of Cardiff fertility knowledge scale show deficiencies in their awareness: a cross-sectional survey in Hunan, China

**DOI:** 10.1186/s12889-020-08937-6

**Published:** 2020-05-29

**Authors:** Yanhui Zhou, Yang Luo, Ting Wang, Yanhui Cui, Mingzhu Chen, Jingxia Fu

**Affiliations:** 1 Xiangya Nursing School, Central South University, 172 Tongzipo Road Yuelu District, Changsha, 410013 Hunan China; 2 The first affiliated Hospital of University of South China, 69 Chuanshan Road Shigu District, Hengyang, 421001 Hunan China; 3 Department of Anatomy and Neurobiology, School of Basic Medical Science, Central South University, 172 Tongzipo Road Yuelu District, Changsha, 410013 Hunan China

**Keywords:** Fertility knowledge, Fertility intention, Childbearing, College students

## Abstract

**Background:**

Fertility knowledge is vital to the fertility health of young people and greatly impacts their fertility choices. Delayed childbearing has been increasing in high-income countries, accompanied by the risk of involuntary childlessness or having fewer children than desired. The aim of this study was to investigate knowledge about fertility issues, the related influencing factors, the method of acquiring fertility knowledge, and the relationship between fertility knowledge and fertility intentions among college students.

**Methods:**

An online cross-sectional survey of Chinese college students was conducted in Hunan Province from March to April 2018. A total of 867 college students from three comprehensive universities responded to a poster invitation utilizing the Chinese version of the Cardiff Fertility Knowledge Scale (CFKS-C). Data were explored and analysed by SPSS (version 22.0) software. Descriptive statistics, chi-squared tests, T-tests, and Pearson’s correlations were used for the measurements.

**Results:**

The average percent-correct score on the CFKS-C was 49.9% (SD = 20.8), with greater knowledge significantly related to living in a city district, being not single status, majoring in medicine, being in year 4 or above of study, and intention to have children (all *p*<0.05). A total of 81.9% of the participants reported that they would like to have children, the average score of the importance of childbearing was 6.3 (SD = 2.7), and the female score was lower than the male score (*p* = 0.001). A small positive relationship was observed between the CFKS-C and the importance of childbearing (*r* = 0.074, *p* = 0.035). Respondents indicated that they gained most of their knowledge from the media and internet (41.4%) and from schools (38.2%).

**Conclusion:**

Yong people in college have a modest level of fertility knowledge, a relatively low intention to have a child, and deficiencies in fertility health education. There is a need to improve the accessibility of fertility health services by developing a scientific and reliable fertility health promotion strategy.

## Background

Fertility is one of the major components of population growth and age structure change and is central to the Programme of Action of the International Conference on Population and Development. The past six decades have witnessed a major decline in world fertility, especially in the developing world [1, 2]. The total worldwide fertility rate fell from almost 5 births per woman in 1950–1955 to 2.5 in 2010–2015 [3]. In many high-income countries, the fertility rate is below the 2.1 babies per woman required for population replacement [4]. According to the latest data released by the National Bureau of Statistics in China, the birth rate in mainland China was 10.9‰ by the end of 2018, a reduction of 1.5‰ compared with 2017 and a reduction of 11.5‰ from 1988 (22.4‰) [5]. Although the “universal two-child” policy has been in place for 4 years, the number of new births and the fertility rate are still declining, raising concerns in many fields of study. Some scholars have predicted that China’s current population situation is similar to that of Japan 20 years ago and that a low birth trap will occur [6]. Similar to changes in total fertility, the age pattern of childbearing and marriage has markedly changed simultaneously. The age pattern of childbearing has been shifting to higher ages, with the mean age at childbirth reaching 30 years or above [7]. In addition, postponing parenthood is associated with an increased risk of higher maternal age and more pregnancy-related complications.

Reasons for low fertility are multifactorial and often difficult to disentangle. The published literature has stated that despite changes in social and economic development, the lack of knowledge about fertility and transformation of fertility concepts are crucial. An international survey of 79 countries from the International Fertility Decision-making Study (IFDMS) showed that the average score on a test of fertility knowledge was 56.9% [8]. Other studies confirmed the poor fertility knowledge and fertility awareness among people of reproductive age in the United States, Sweden, Germany, Italy, Japan and other countries [9–15]. In addition, some studies have demonstrated that some professionals, including obstetrics and gynaecology resident physicians and nurses, tend to underestimate age-related fertility decline and overestimate the success rate of fertility treatments [16–19]. Compared with Western countries, fertility knowledge is relatively less documented in Asia. A cross-sectional study conducted in Thailand revealed a considerable knowledge gap about the factors that influence fertility in reproductive-age individuals (males and females aged 18–45 years) [20]. Japanese professor Maeda found that fertility knowledge was low [13], and women with fertility knowledge gave birth to their first child 2.34 years earlier compared with those without such knowledge [21]. Thus, fertility knowledge will affect personal fertility choices or intentions.

In prior studies, college students, as a particular group of young people, have been in focus given that they are of reproductive age and face choices between education and career versus marriage and childbearing. Previous surveys among young college students in Sweden, Italy, America, and New Zealand revealed that knowledge about human fertility was rather poor [15, 22–24]. In China, the proportion of the population with a college education or above has increased from 2.8% in 1998 to 13.9% in 2017 [6]. However, little is known about the level of fertility knowledge among Chinese mainland college students. Moreover, fertility health education and sexual education are implicit on account of Chinese traditional culture; related courses are rarely provided and are relatively underdeveloped due to the lack of teachers and appropriate textbooks [25–28], and there is little research on how students acquire fertility knowledge.

Therefore, the aim of the present study was to investigate the level of fertility knowledge among college students in Hunan Province, the related influencing factors, the methods by which students acquire fertility knowledge, and the relationship between fertility knowledge and fertility intention.

## Methods

### Design

A cross-sectional survey of college students was conducted in Hunan Province, China, during a one-month period of March 8 to April 8, 2018.

### Sample and setting

The survey was conducted in Hunan Province, China, which is located in the middle of China. In this province, student enrolment in regular institutions of higher education is 22.1 persons per 10,000 population, and there are 109 schools that are regular institutions of higher education [29]. Three comprehensive universities were selected by convenience sampling. Each university has a population of approximately 30,000 students who come from different provinces in China.

Inclusion criteria: 1) Aged between 18 and 30 years old; 2) Full-time college students of regular institutions of higher education; 3) Informed consent and voluntary participation. Exclusion criteria: 1) Non-Chinese student; 2) Pregnant; 3) Have children. Students were recruited by open recruitment as advertised by poster. These posters, including a brief description of the study and a QR code, were posted on the notice boards of each university, canteen and dormitory in the university to be seen by all students and staff. By scanning the QR code using a mobile phone, students could obtain a brief description of the study, an informed consent form and the questionnaire (Questionnaire star software). The questionnaire star software (Ranxing information technology company, Changsha, China), the first and largest domestic online questionnaire survey and test platform, has sent more than 24.6 million questionnaires and collected more than 1.6 billion questionnaires from respondents. The online survey was open from March 8 to April 8, 2018. Participants who completed the questionnaire were provided a reward incentive via the internet.

The sample size was estimated by the formula *n* = *u*_α/2_^2^π(1 − π)/*δ*^2^ Uα/2 = 1.96, δ = 0.05, π = 0.569 [8], put into the formula, the calculated sample size was about 376. In order to avoid errors, the sample could be expanded by 10% ~ 20%, 376 ~ 451. In total, 867 individuals completed the online questionnaire during the 1-month collection period, and 799 questionnaires were valid.

### Questionnaire and study variables

The questionnaire consisted of 25 items covering 4 domains. It was pilot tested twice with 30 college students to ensure that the instrument was understandable. The pilot data were not included in the analysis.

#### Sociodemographic variables

Participants stated their age in years, gender, place of birth, whether they were of the only child generation (yes/no), sexual orientation, relationship status, their discipline, and the year of study.

#### Intentions of childbearing

One question investigated the personal intention to have children (yes/no), and one 0- to 10-point response scale surveyed the personal perceived importance of childbearing [15].

#### Fertility knowledge

The Chinese version of the Cardiff Fertility Knowledge Scale (CFKS-C) was translated into Chinese from the original Cardiff Fertility Knowledge Scale (CFKS) developed by Boivin et al. (2013) and shown to have satisfactory validity and reliability. The CFKS consists of 13 items. Three areas of knowledge about fertility are measured: risks for reduced fertility, misconceptions about fertility, and basic facts about infertility. A three-point scale of “true”, “false”, or “do not know” was used to rate all items. One point was given for a correct answer, and zero points were given for an incorrect or unknown answer. Scores were reported as percentages that were equal to points divided by the total number of questions. In the original CFKS, the internal consistency coefficient alpha (Cronbach’s α) was 0.79 [8]. The CFKS-C was developed through Brislin’s translation/back-translation method and modified through cultural adaptation and semantic analysis. The Cronbach’s α coefficient was 0.827, the test-retest reliability was 0.826, and the Scale-Content Validity Index (S-CVI) was 0.950 with the Item-Content Validity Index (I-CVI) ranging from 0.875 to 1.000 [30].

#### Fertility health education

In addition, three questions were used to investigate students’ perceived knowledge of fertility issues: (I) their perceived education level of fertility-related knowledge (not at all educated, somewhat educated, educated, highly educated), (II) whether or not they discussed fertility issues with family (yes/no), and (Ш) where they gained most of their knowledge about fertility issues (media and internet, schools, family, friends, doctors/gynaecologists, non-government organizations, other).

### Data analyses

Descriptive statistics were used to describe the sociodemographic variables and fertility knowledge. Categorical data were compared using Chi-squared tests. T-tests and analysis of variance (ANOVA) were used to compare the total scores on the CFKS-C between sociodemographic categories. Pearson’s correlation was used to examine the association between the importance of childbearing and the CFKS-C score. The data were assessed for errors before double-entry computer input. Statistical significance was defined as a two-sided *P* value less than 0.05. All analyses were performed using SPSS (version 22.0) software (IBM Corp., Armonk, New York, USA).

### Ethics statement

Ethical review and approval were performed by the IRB of behavioural and nursing research in the School of Nursing of Central South University (Project Number 2017028). Permission to use the CFKS was obtained from the authors who developed it. A cover letter was presented to respondents to explain the aim and process of the study before the questionnaire was shared. After participants read the contents of the consent form and agreed to participate in the study, they checked “consent” button and began to fill in the questionnaire. Participation in this study was voluntary, anonymous and confidential. To maintain anonymity, participants were asked not to provide their name or telephone number. Collection of online data complied with the “Code of Conduct of Marketing Research”. All data collected were treated anonymously and confidentially.

## Results

### Participants’ characteristics

The average age of respondents was 19.7 (SD = 2.0), ranging from 18 to 30 years old. Female participants accounted for 56.8% of the sample, and 68.8% of participants were single. The sociodemographic characteristics of the participants are summarized in Table 1.

**Table 1:** Sociodemographic characteristics of participants (*N* = 799)

Characteristics		Total (*N* = 799)	Male (*N* = 345)	Female (*N* = 454)
Age (years)	Mean (SD)	19.7 (2.0)	19.6 (1.8)	19.8 (2.1)
	Range	18 ~ 30	18 ~ 28	18 ~ 30
		n (%)	n (%)	n (%)
Place of birth	City district	332 (41.6)	155 (44.9)	177 (39.0)
	Not in the city district	467 (58.4)	190 (55.1)	277 (61.0)
Only child	Yes	251 (31.4)	130 (37.7)	121 (26.7)
	No	549 (68.6)	215 (62.3)	333 (73.3)
Sexual orientation	Heterosexual	733 (91.6)	313 (90.7)	419 (92.3)
	Homosexual	15 (1.9)	11 (3.2)	4 (0.9)
	Bisexual	52 (6.5)	21 (6.1)	31 (6.8)
Relationship	Single	549 (68.7)	243 (70.4)	306 (67.4)
	Non-single	250 (31.3)	102 (29.6)	148 (32.6)
Discipline	Non-medicine	525 (65.7)	249 (72.2)	276 (60.8)
	Medicine	274 (34.3)	96 (27.8)	178 (39.2)
Year of study	1	416 (52.0)	204 (59.1)	212 (46.7)
	2	246 (30.8)	95 (27.5)	151 (33.2)
	3	66 (8.3)	22 (6.4)	44 (9.7)
	4 or above	71 (8.9)	24 (7.0)	47 (10.4)

### Fertility knowledge and influencing factors

The average percent correct score on the CFKS-C was 49.9% (SD = 20.8). Table 2 shows the fertility knowledge score for each item and the rank order.

**Table 2:** The percentage of participants who answered each item correctly (*N* = 799)

Items *(correct answer)*	Male (*N* = 345)	Female (*N* = 454)	Total *N* (%)	Rank
A woman is less fertile after the age of 36 years. *(true)*	257 (74.5)	375 (82.6)	632 (79.1)	1
A couple would be classified as infertile if they did not achieve a pregnancy after 1 year of regular sexual intercourse (without using contraception). *(true)*	133 (38.6)	143 (31.5)	276 (34.5)	10
Smoking decreases female fertility. *(true)*	245 (71.0)	362 (79.7)	607 (76.0)	2
Smoking decreases male fertility. *(true)*	252 (73.0)	344 (75.8)	596 (74.6)	3
Having a healthy lifestyle makes you fertile. *(false)*	20 (5.8)	10 (2.2)	30 (3.8)	13
About 1 in 10 couples are infertile. *(true)*	108 (31.3)	165 (36.3)	273 (34.2)	11
If a man produces sperm he is fertile. *(false)*	227 (65.8)	342 (75.3)	569 (71.2)	4
These days a woman in her 40s has a similar chance of getting pregnant as a woman in her 30s. *(false)*	226 (65.5)	331 (72.9)	557 (69.7)	5
If a man has had mumps after puberty he is more likely to later have a fertility problem. *(true)*	93 (27.0)	126 (27.8)	219 (27.4)	12
A woman who never menstruates is still fertile. *(false)*	158 (45.8)	251 (55.3)	409 (51.2)	7
If a woman is overweight by more than 2 stone (13 kg or 28 pounds) then she may not be able to get pregnant. *(true)*	120 (34.8)	156 (34.4)	276 (34.5)	9
If a man can achieve an erection then it is an indication that he is fertile. *(false)*	192 (55.7)	227 (50.0)	419 (52.4)	6
People who have had a sexually transmitted disease are likely to have reduced fertility. *(true)*	146 (42.3)	169 (37.2)	315 (39.4)	8

As shown in Table 3, the CFKS-C scores were significantly higher in participants born in city districts, currently being not single status, participants in the medicine discipline, and participants in year 4 or above.

**Table 3:** Univariate analysis for factors related to fertility knowledge

Variables (Sociodemographic)		CFKS-C^#^ (Mean + SD)	T/F value	*p*-value
Gender	Male	48.5 (22.9)	−1.514	0.131
	Female	50.8 (19.0)		
Place of birth	City district	52.4 (20.4)	2.910	0.004*
	Not in the city district	48.1 (20.9)		
Only child	Yes	48.7 (21.4)	−1.064	0.288
	No	50.4 (20.5)		
Sexual orientation	Heterosexual	49.7 (20.8)	1.223^#^	0.295
	Homosexual	45.6 (23.7)		
	Bisexual	53.7 (19.2)		
Relationship	Single	48.6 (20.1)	−2.565	0.011*
	Non-single	52.7 (21.9)		
Discipline	Non-medicine	46.8 (20.9)	−5.887	0.000*
	Medicine	55.72 (19.4)		
Year of study	1	47.1 (20.8)c	6.8576^#^	0.000*
	2	52.1 (20.9)b		
	3	50.4 (19.0)b		
	4 or above	57.6 (19.6)a		

*CFKS-C* Chinese version of the Cardiff Fertility Knowledge Scale

^#^ the average percent correct score on the CFKS-C

T/F value: the value of T-tests and analysis of variance (ANOVA)

* *p* < 0.05; SD, standard deviation; ^#^, F value

a, b, c: different letters at the end of figure on the same column indicate significant differences(*P* < 0.05)

### Intention of childbearing

Among participants, 81.9% said that they would like to have children, while 18.1% reported that they had not thought about it. The CFKS-C scores (mean = 50.6%, SD = 20.7) of those who intended to have children were recorded, and these scores were significantly higher than the scores of those who did not intend to have children (mean = 46.6%, SD = 21.2) (t = 2.066, *p* = 0.039). When participants were asked to rate the importance of childbearing on a scale of 0–10, the average score was 6.3 (SD = 2.7). Female’s scores (mean = 6.1, SD = 2.7) were lower than male’s (mean = 6.7, SD = 2.7) (t = 3.453, *p* = 0.001). A small positive relationship was observed between CFKS-C and the importance of childbearing (*r* = 0.074, *p* = 0.035).

### Fertility health education

Furthermore, 67.7% discussed fertility issues with family. More than half (54.2%) of the participants reported that they were “somewhat educated” about fertility issues, followed by “educated” (30.9%) and “not educated at all” (10.4%). Only 4.5% of the participants stated that they were “highly educated”. Respondents stated that they gained most of their knowledge from the media and internet (41.4%), schools (38.2%), family (9.1%), friends (6.6%), doctors/gynaecologists (2.9%), and non-government organizations (1.5%).

## Discussion

### Low fertility knowledge

The current study found that young college students in China (mainland) had a modest level of fertility knowledge. This finding was consistent with the findings of previous studies [8, 13]. The IFDMS reported that the average score was 56.9% correct in 79 countries but less than 44.9% in China, and the average score was 59.7% in the university-educated population [8]. In this study, although all participants were college students, the average score was only 49.9%, higher than the previous study in China, lower than the sample of university education population in 79 countries [8] and in Japan [13]. In general, greater fertility knowledge was associated with higher education [8, 9, 21, 31]. It follows that fertility knowledge remains low in China.

The findings of the present study also indicated that most of the participants had misconceptions about fertility and the basic facts of infertility. Table 2 reveals that college students underestimate the incidence of infertility and the influence of mumps and overestimate the importance of a healthy lifestyle to fertility. In fact, more than half of participants had misperceptions about fertility, although 89.6% reported that they were educated about fertility issues. This may have led to delayed childbearing and to neglecting some risk factors for reduced fertility. In regard to health issues, based on explanatory models for behaviour change, lack of knowledge is the core reason why people do not behave optimally [8].

All participants with medicine discipline had increased fertility knowledge, as did those who were female, born in a city, those with non-single status, those being in year 4 or above of study, and those with an intention to have children. These findings are consistent with previous studies, which reported that greater fertility knowledge was associated with female gender [8, 13, 22], socioeconomic status [8, 13] and with medical consultation for infertility [8]. These may be caused by the persistent lag in fertility health education and by cultural differences. Many people are aware of the necessity of sex education, but they feel embarrassed when discussing sex or fertility issues; this is especially true in unmarried populations [21, 22]. Moreover, a positive relationship between education and health literacy has been recorded in hypertension, diabetes, and HIV health contexts [32–35]. Therefore, it is urgent to improve fertility health education in China, and it is particularly important to consider those items and populations that had low scores. Targeted promotion of fertility knowledge may raise awareness, improve uptake of more effective methods and improve fertility decision-making in certain population groups [36].

### Low intention to have children

Approximately one-fifth (18.1%) of the respondents stated that they did not want to have children, which was higher than previous studies in the US (14.2%) and Sweden (4.1%) [15, 23] and slightly lower than the sample in Hong Kong (19.1%) [31]. When indicating how important childbearing was to them, both male and female college students rated it as highly important (mean = 6.3, SD = 2.7), and the score was lower than those found in Denmark (male: mean = 8.2, SD = 3.1; female: mean = 9.7, SD = 2.3) [11] and the US (male: mean = 8.3, SD = 2.5; female: mean = 8.5, SD = 2.9) [23] but higher than in Hong Kong (mean = 6.2, SD = 3.0) [37]. These findings are in line with the fertility rate in China. This phenomenon may result from the country’s fertility policy of the last 30 years. The two-child policy has been implemented for 4 years in China; however, an increasing number of newlyweds put off having children or decide not to have children at all to pursue higher education or career development. A previous study demonstrated that providing fertility-related information contributes to greater reproductive knowledge and may affect childbearing intention [38]. Furthermore, males had significantly higher scores than females, a finding that was contrary to other results [12, 15, 23, 37]. Explanations for this may be that women – especially highly educated women – must balance childbearing with education, career aspirations, health and partner selection. Previously, published literature found that delaying childbearing has steadily increased in recent decades [39, 40]. Modern women are independent and pay more attention to their development and freedom than did women of earlier generations; having children means more effort and burden [41]. Furthermore, an increasing number of women identify with feminism [42].

Moreover, this study found a small positive relationship between CFKS-C and the importance of childbearing. Therefore, improving fertility knowledge may be beneficial for increasing the fertility rate.

### Lack of corrected fertility health education

Overall, more than three-quarters of participants self-reported that they had some education about fertility-related knowledge, and more than half discussed fertility issues with family, but their rate of correct fertility knowledge was actually low. Furthermore, the findings showed that more than half of college students obtained knowledge from media and the internet (41.4%) instead of school and the health department. However, in the US, the main modes of acquiring fertility knowledge were school (46.0%) and family (20.0%) [23]. These findings may reveal that young people have an incomplete understanding of fertility issues, and they seek fertility information in a haphazard way from less formal sources. The World Health Organization (WHO) recently established the goal of supporting sexual and reproductive health, including fertility care [43]. Hence, fertility health promotion strategies should be developed. First, public health initiatives should set up a special organization to be responsible for fertility education. For example, in 2011, a government-funded fertility health promotion programme was established in Australia to increase fertility awareness [44]. Second, education about fertility should be strengthened in the school and primary health care setting [45, 46]. Considering that teachers and primary health care professionals may be suitable candidates to deliver health information about fertility, they should be educated with additional information and resources [10, 47]. Third, the forms and channels of publicity about fertility should be broadened, and information should be standardized, especially on the internet. Internet education is a double-edged sword, and both accurate and inaccurate knowledge can be spread quickly. However, given that information from the internet can be obtained easily and conveniently, web-based approaches can be applied to fertility education. Previously, published literature has demonstrated that a short educational intervention (including online education) was effective in increasing knowledge about reproductive health and fertility, thus supporting family planning decisions [31, 48–50]. But, fertility information may increase infertility threats and feelings of anxiety [50, 51]. Given these issues, future research should focus on developing and evaluating interventions to improve knowledge about fertility health and to investigate the barriers and enablers of fertility health education.

### Limitations

**First**, the cross-sectional design of the present study had self-selection bias, which was inherent in using the convenience sampling method.

**Second**, the response rate and the characteristics of the non-respondents could not be ascertained because the participants were recruited by posters at the university.

**Third**, the population in three universities in Hunan province may not reflect the fertility knowledge level in other universities of Hunan province or other parts of China when generalizing the findings.

**In addition**, to collect more credible and comprehensive information, both quantitative and qualitative methods could be used in the future.

## Conclusions

This study reveals that college students have a modest level of fertility knowledge, a relatively low intention to have a child, and deficiencies in fertility health education. There is a need to improve the accessibility of fertility health services by developing a scientific and reliable fertility health promotion strategy. Because fertility decisions should be based on correct and complete information, educational institutions and providers need to take action to embed fertility education into curricula for college students and the general public.

## Data Availability

The datasets used and/or analysed during the current study are available from the corresponding author on reasonable request.

## Abbreviations

CFKS: Cardiff fertility knowledge scale
CFKS-C: The Chinese version of Cardiff fertility knowledge scale
IFDMS: International Fertility Decision-making Study
SD: Standard deviation
